# From Policy to Practice: Measures Against Sexual Abuse by Swedish Sports Federations

**DOI:** 10.3389/fspor.2022.841653

**Published:** 2022-03-02

**Authors:** Susanne Johansson

**Affiliations:** The Swedish School of Sport and Health Sciences, The Institution of Movement, Culture and Society, Stockholm, Sweden

**Keywords:** sexual abuse, sexual harassment, violence, safe sport, child protection, athlete welfare, sport policy

## Abstract

The sport movement must protect children and young athletes from all forms of abuse. However, research points to a disconnect between policy and implementation of policy against sexual abuse. No studies have investigated measures against sexual abuse in Swedish sport. The purpose of this study was to explore measures against sexual abuse in the 10 largest sports federations (SFs) for child and youth sport in Sweden. The study draws on interviews with representatives (*n* = 18) of the SFs and on a review of SFs' website content regarding sexual abuse and safe sport. Results show that the SFs have taken few or no measures against sexual abuse. Measures for safe sports vary in existence, development, and organization between the SFs, and many SFs are in the early stages of safe sport measures and practice. Although the SF representatives emphasize that sexual abuse is unacceptable, a conflict between making it visible or invisible emerges and creates a gap between policy and practice. Reproducing a culture of silence around sexual abuse in sports seems advantageously for SFs. Social and organizational factors that can debilitate safe sport measures and facilitate sexual abuse in sport are discussed.

## Introduction

Sexual abuse involves all forms of unwanted and abusive sexual behaviors. It is a global social problem that can severely harm the lives and health of those subjected to it (Stoltenborgh et al., 2011). Sport is by no means immune to sexual abuse (e.g., Auweele et al., 2008; Fasting et al., 2011; Mountjoy et al., 2016; Parent et al., 2016; Vertommen et al., 2016; Johansson and Lundqvist, 2017). Elite-sport in particular has been associated with vulnerability and risk of abuse, including sexual abuse of young athletes (Wilinsky and McCabe, 2020). Sexual abuse violates children's rights and human rights as well as the foundational values and policy of sport on national and international levels (UNICEF, 2010; Mountjoy et al., 2015; BRIS, 2017).

The risks and harm of sexual abuse are affected by the inclination to disclose abuse and the kind of help and support that is offered—and reaches—those in need of it (Parent, 2011). Unfortunately, the literature reveals a history of ignorance and silence of sexual abuse in sport and governing agencies in sport rarely acknowledge or address sexual abuse (e.g., Parent and Hlimi, 2013; Brackenridge and Rhind, 2014; Kerr et al., 2014; Lang and Hartill, 2014; Mountjoy et al., 2016). Studies as well as sexual abuse cases reported in the media indicate that safeguarding measures in sports often fall short in protecting athletes (Parent and Demers, 2011; Mountjoy et al., 2016; Johansson and Lundqvist, 2017; Bjørnseth and Szabo, 2018; Kerr et al., 2020). In response to the many public scandals of sexual abuse in sport and 30 years of research emphasizing this as a prevalent problem in sport, safe sport policy and procedures have been developed (Rhind and Owusu-Sekyere, 2020). Correspondingly, this development has been the focus of a growing number of studies into safeguarding and child protection in sport (Brackenridge and Rhind, 2014). It has been found that few protection measures are evidence-based or built on theory, and they are rarely evaluated to determine whether the intended outcomes have been achieved (Parent and Hlimi, 2013; Hartill and O'Gorman, 2014; Lang and Hartill, 2014; Gurgis and Kerr, 2021). According to the literature, the implementation of policy has not kept pace with policy development, which created a problematic gap between policy and practice (Brackenridge, 2002; Parent and Demers, 2011; Donnelly et al., 2016; Rulofs et al., 2019; Kerr et al., 2020).

Bridging the gap between policy and practice is crucial for developing safe sport environments and preventing abuse at all levels of sport (Lang and Hartill, 2014). As UNICEF emphasized in their report about protecting children from violence in sport: “Good intentions and written policies mean nothing if they are not translated into action” (2010, p. 21). The circumstances and state of the safeguarding of sport demonstrate a need to align research, policy, and practice for the development of safe sport measures. Parent and Demers (2011) suggest that such alignment could facilitate empirically informed measures and the development of a theoretical foundation to inform practice and to develop systemic, sustainable safe sport measures and a safe sport culture. Evaluation is an important step in the development of evidence-based policy and practice. Vitally important knowledge can be gained by examining safeguarding and protection measures in sport, which in turn will help improve the implementation of policy (Parent and Hlimi, 2013; Hartill and O'Gorman, 2014).

The present study is an attempt to contribute to the implementation of sport policy by exploring measures against sexual abuse in sport in Sweden. Previous reports have noted that Swedish sport is lagging when it comes to athlete safeguarding compared to other European countries (Chroni et al., 2012; Mergaert et al., 2016). Research into sexual abuse in sport in Sweden has been sparse and no studies have explored measures against sexual abuse in Swedish sport (Johansson, 2017). The purpose of this study was to explore measures against sexual abuse in the ten largest sports federations (SFs) for child and youth sport in Sweden. Swedish child and youth sport include athletes between the ages 7 and 25years and disabled athletes of all ages. Representatives from the SFs were interviewed about their measures against sexual abuse and their experiences with safe sport and sexual abuse cases. The following four research questions were explored:

1. Which measures against sexual abuse have been implemented by the SFs?
2. How is sexual abuse addressed on the SFs website?
3. How is the management of reported sexual abuse cases described?
4. Which barriers and facilitators can affect the SFs implementation of sport policy against sexual abuse?

The definition of “sexual abuse” in this study draws on the policy against sexual abuse of the Swedish Sport Confederation (SSC), which broadly applies to all SFs, district federations, and local sport clubs. Sexual abuse is defined as “all sexual actions that are made toward someone, in front of someone, or that one person makes someone else to do, against the subjected person's will” (SSC, 2011, p. 2). The study focusses specifically on the child and youth sport context (i.e., athletes 7–25 years old). I thus use the term “sexual abuse” instead of “child sexual abuse” in this paper to include persons both under and above age 18. I will finish this introduction by describing the context of Swedish sport and sexual abuse.

The sport movement in Sweden as in other Nordic countries has a strong connection to civic society informed by a “sport for all” vision. The Swedish sport movement is governed by The Swedish Sports Confederation (SSC). The SSC is the national umbrella organization of Sweden's 72 national sport federations (SFs). In turn the sport movement contains approximately 800 district federations and 20,000 local sports-clubs. The governmentality of Swedish sport is informed by relative autonomy between the SSC, SFs, district federations, and sport-clubs (Barker-Ruchti et al., 2018). Moreover, Swedish sport is primarily organized on a voluntary, non-profit, government funded basis. The majority of leaders, directors, and coaches in Swedish sport-clubs are volunteers (Eliasson, 2017). The SSC administers government funding to the SFs. To receive this funding, sport organizations must, among other things, implement a “child rights perspective” (SSC, 2009).

During the last decade, sexual abuse of children and young athletes in sport has received increased attention in Sweden as in many other countries. Sweden's first high profile case was reported in 2011 when retired high-jumper Patrik Sjöberg (Olympic medallist 1984, current European record holder, and ranked third in the world to this day) revealed that his former coach had sexually abused him and several other young male athletes. During the #metoo movement in 2017 and 2018, sexual abuse received massive public attention and debate in Sweden as in many other countries. That debate and the numerous testimonies also included the sport context under the hashtag #timeout. Since then, the culture of silence in sport has been a reoccurring topic in public debate and news about abuse in sport. For example, the Kyle Beach disclosure in 2021 about sexual abuse in the NFL sparked a wave of testimonies of abuse that had gone unreported in Swedish ice hockey and other sports (e.g., Dagens Nyheter, 2021).

In response to the attention to sexual abuse in Swedish sport, especially in 2011 and onwards, there has been somewhat of a shift from promoting foundational values and benefits of sport to child protection and prevention of harm (cf. Gurgis and Kerr, 2021). “Safe sport” developed as the overarching phrase during this shift. Safe sport measures initiated by the SSC involve a national code of conduct for the sports movement, a policy against sexual abuse, and written guidelines for the safeguarding of children in sports. The first policy document against sexual harassment in sport (SSC, 2005) emphasizes the responsibility to act according to policy:

Considering that sport is the greatest popular movement in Sweden and by far the largest child and youth organization we have an obvious responsibility to actively prevent and counter sexual harassment. All forms of harassment contravene the mission statements of sport. (SSC, 2005, p. 3)

In 2011 an additional policy document against sexual abuse was adopted (SSC, 2011). This policy specifically addresses the protection of children and young athletes from sexual abuse and outlines prevention and intervention measures that broadly applies to the Swedish sport movement. In addition to these policies and guidelines, the SSC has established a whistle-blower function and a Sports Ombudsman. Also, since 2020 criminal record checks of persons working, paid or unpaid, with children in sports is mandatory. Thus, measures to detect, report, and prevent potential misconduct and harm, including harassment, abuse, bullying, violence, and neglect have been developed by the SSC.

## Materials and Methods

### Theoretical Framework

This is a study of sport as a social and cultural institution. From a sociological perspective, safe practice in sport environments can prevent sexual abuse and protect athletes. However, socialization can also enable sexual abuse by normalizing abusive behaviors and power imbalance on individual, relationship, organizational, and structural levels (Johansson, 2017; Rhind and Owusu-Sekyere, 2020; Roberts et al., 2020). Power inequity operates on all levels in society and sports. In their systemic review of organizational factors and non-accidental violence in sport, Roberts et al. (2020) identified organizational tolerance as the first and power imbalance as the second key factors associated to sexual abuse. Organizational tolerance for abuse informed by the status quo of sport can create a culture of silence that contributes to athlete isolation and to the camouflaging of sexual abuse.

Organizational factors that can facilitate sexual abuse and debilitate safeguarding measures in sport likely apply to the Swedish sport movement. Due to factors such as autonomy, self-governance, limited financial and human resources, and voluntary staff, Swedish sport may be ill-equipped to prevent sexual abuse and governing federations to enforce desired practices (cf. Parent and Demers, 2011; Donnelly et al., 2016; Eliasson, 2017; Barker-Ruchti et al., 2018; Roberts et al., 2020). Drawing on this theoretical framework, developing a safe culture in sport requires an integrated whole-of-systems approach involving individual agency, social structure, and organizational investment (Rhind et al., 2017; Rhind and Owusu-Sekyere, 2020; Roberts et al., 2020). In the present study, these measures equate to the implementation of policy against sexual abuse through practical measures taken by people within Swedish SFs. SF representatives can provide insights into safeguarding measures and the ideas, knowledge, and perceptions that inform them. Their accounts can contribute to an understanding of the socio-cultural structures and organizational factors that can facilitate or debilitate measures against sexual abuse.

### Sample

This study involves an examination of measures against sexual abuse taken by Swedish sport federations (SFs). The study was funded by the SSC. A purposive sample was decided on in collaboration with the SSC to include the 10 largest SFs.^1^ The selection of SFs was based on state activity funding for child and youth sports (LOK-stöd). In other words, SFs with the most sport activities during the year of 2018 for athletes between 7 and 25 years of age and physical or intellectual disabled athletes of all ages were included. The sampled SFs manage the following sports on a national level: athletics, basketball, equestrian sports, floorball,^2^ football, gymnastics, handball, ice hockey, and swimming. Some SFs represent one sport (e.g., the Swedish Basketball Federation), others more than one (e.g., the Swedish Swimming Federation, which covers swimming, artistic swimming, diving, and water polo). Together, the 10 sampled SFs organize the vast majority of the child and youth sports in Sweden. These SFs thus have a significant impact on clubs and young athletes.

The sampling and recruitment of participants included two primary steps. First, managing directors of SFs were emailed about the study. The initial intent was to recruit two representatives from each SF: one SF representative with a general officer or management position and one with more particular responsibilities for safe sports. These sampling criteria were presented in the initial e-mail and the directors were asked to suggest potential participants, including themselves, who met these criteria. However, most SFs did not have positions or staff exclusively committed to safe sports and I was unable to recruit two representatives from every SF who met these sampling criteria. Instead, I adapted the sample to the positions and responsibilities available in each SF. Thus, only one participant represented the SFs for tennis and floorball. Second, potential participants were contacted individually with detailed information about the study and an invitation to participate. The final sample included 18 participants. They held positions in their SF as secretary general, sports director/chief executive, child and youth sports officer, development officer, sport/member ombudsman, board member, and safe sport council representative. Some participants had worked in their SF for many years (and were once athletes in the sport themselves), others were quite new in their position or the SF.

### Materials and Procedures

The study draws on two data sets. Interviews with the sampled SF representatives (*n* = 18) were the primary source of data. In addition, a review of the SFs website content on sexual abuse and safe sport was performed. Below, each of the procedures are described, including the data collection and analysis.

#### Interviews

A qualitative method was used to conduct interviews to explore measures (or lack thereof) against sexual abuse by the SF and gain an understanding of SFs representatives' experiences with safe sport and management of sexual abuse cases. The interviews took place between November 2019 and February 2020. I handled all interview procedures, including participant interaction, interviewing, data transcription, and analysis. The interviews took place face-to-face in private locations (typically conference rooms) at the SFs' offices, chosen by the participants. Prior to the interviews, we talked about the study and participants were invited to ask questions. Written consent forms were signed by participants prior to the interviews. As interviewer my role was to facilitate the conversation by asking questions, listening actively, and being responsive (Bryman, 2016). A semi-structured interview guide with thematically organized topics relating to the research questions was used. The interview themes included (1) background, (2) SFs policy and guidelines regarding sexual abuse, (3) prevention and intervention measures, (4) case management, (5) facilitators and barriers. Probes were used to gain detailed accounts from the SFs-representatives. Interviews lasted between 1 and 3 h and were digitally recorded. The interviews were transcribed verbatim, except for details that were altered for the sake of confidentiality (e.g., names, sport events, and locations).

The study's research questions guided the analysis by outlining three main themes: (1) measures against sexual abuse, (2) experiences of case management, (3) facilitators and barriers to the implementation of sport policy against sexual abuse. First, an initial coding into the three themes was performed. During the second coding the following subthemes were labeled:

1) Measures: (a) existence of measures, (b) policy and guidelines, (c) criminal record checks, (d) coach licensing, (e) disciplinary bodies, (f) Ombudsman functions, (g) education, (h) case management
2) Experiences of case management: (a) incidence of reported cases, (b) experience of case management
3) Barriers and facilitators: (a) facilitators, (b) barriers

Lastly quotations that illustrated each subtheme was highlighted. The results were organized and presented with the themes and subthemes as outlined. I translated the quotations to English.

#### Review of Sport Federation Websites

Young people often turn to the Internet for information (Kerr et al., 2014); SF websites may thus be an important source for educating, displaying, and normalizing the conversation about sexual abuse, as well as for sharing directions, resources, and support. For example, website content can contain information that is directly addressed to children and young people, which may help to empower and protect them against sexual abuse (Eliasson, 2017; Hartill et al., 2019). The purpose of reviewing the SF websites was to gain insight into their display and content about sexual abuse and safe sports. It should be acknowledged that website content does not necessarily mirror SFs protection measures more broadly, inaccessibility of information on websites may be a question of functionality of and (poor) web-design rather than actual content. Moreover, the data collection was performed during the first stage of the study, in October 2019. It is likely that the websites have been updated since data collection. The SFs representatives did not know about the website review beforehand to avoid bias (they were informed later on). A checklist was used for the content analysis of the websites (see Table 1, translated by me).

**Table 1:** Checklist for SF websites review.

**Question**	**Examples**
Visibility and display?	How many clicks are required to find content about sexual abuse, sexual harassment or safe sports? Is the content easily accessible?
Search function hits?	Search phrases: sexual abuse, sexual harassment, safe sport
Type of content?	Sport policy, guidelines, and other regulatory documents, educational materials, contact details, web-links for child protection agencies or helplines
Topic areas related to sexual abuse?	Child sexual abuse, sexual or gender harassment, grooming, #metoo, sex-crime, sexual consent, gray areas, and ambiguous boundaries
What perspectives on sexual abuse and/ safe sport can be identified?	Childs rights, child perspective, gender equality, LGBTQ, athlete empowerment
How clear and direct is the terminology?	What terms and language are used? How explicit is the content and information about sexual abuse?
To whom is the content directed?	Victims/survivors of sexual abuse; parents or other adults, children, youth, whistle-blowers; athletes, coaches, sport leaders, directors, others

### Research Ethics and Quality

Ethical considerations need to be prioritized when studying sensitive topics such as sexual abuse for ethically sound research, and also for the quality and trustworthiness of the research (Lee, 1993). Sexual abuse may be an uncomfortable conversation in general. It can be delicate because every case of abuse is a failure to protect young athletes. Thus, inactions and a lack of protection measures can be discrediting to SFs. This raises concerns about honesty, disclosure, and social desirability bias (Lee, 1993; Gurgis and Kerr, 2021). Considerations to meet ethical and quality criteria in the study included the following measures.

The study protocol was reviewed and cleared by the Swedish Ethical Review Authority (Number 2019-04960). For consistency, a trustworthy connection with the participants, and to avoid compromising influences I handled all participant interactions, conducted the interviews, and transcribed the data (Mero-Jaffe, 2011). My prior research experience of conducting interviews on sexual abuse in sport facilitated interaction and trustworthiness. As sole researcher and author though, I have tried to be transparent and to carefully describe the applied methods and procedures to participants and in the paper. Detailed information about the nature and purpose of study, the interview procedure, publication, and ethical considerations was presented to participants in writing. All SF representatives contacted agreed to participate, and many expressed that this research is important. Consent was obtained from the participants verbally and in a writing. Before the interviews started the participants were told they could terminate or pause the interview at any time and that all topics and questions were optional. We discussed the precautions taken to prevent identification of the participants and their position in the SF, including the anonymisation of interview transcripts. Moreover, we talked about the sensitivity of the topic and yet the importance of not shying away from it. This discussion served to facilitate open and honest conversations.

When transcribing the interviews, each SF was assigned a number to minimize the risk of identification of SF or individual SF representatives. In a few cases though, SFs are identified (but no person named) to offer specific examples of safe sport measures. The participants in question were notified and given a chance to review and consent to the quotes before they were included. When investigating measures against sexual abuse it is likely that SF representatives feel scrutinized and anxious to depict their work and their sport in a favorable and responsible manner to some extent. Despite these considerations regarding ethics, quality, and trustworthiness, it is possible that the SF representatives were anxious to depict their sport and their work in this area in a favorable and responsible manner. Although the study is not based on data triangulation, I believe the SF website review was useful to include another data source in addition to the interviews. In a few cases, the SF representatives referred to their websites themselves for further information about the measures they described during the interviews.

## Results

The results are presented in the following order: Measures against sexual abuse, including website content review, experiences with case management, and facilitators and barriers for implementing sport policy against sexual abuse.

### Measures Against Sexual Abuse

Safe sport is a developing area in Sweden. At the time of data collection, five of the 10 SFs (the athletics, basketball, football, handball, and swim SF) had received funding from the SSC for safe sports and had initiated an organizational foundation and structure for safe sport measures. Many SFs are thus taking initial steps toward this.

SFs have no specific measures to target sexual abuse (e.g., grooming or sexual consent and sexual relations in relationships of power, trust, and dependency in sport). It was also uncommon for the SF to address sexual abuse openly and explicitly. Thus, the development of safe sport measures can be seen as a process in which sexual abuse prevention is not among the first steps:

We have no specific measures to protect against sexual abuse. Thus far, we haven't had any platform to build upon, so it's a bit strange to focus exclusively sexual abuse. (SF3)

We must establish a structure, create guidelines if something happens … we have no such structure yet. We don't really inform about it, none of that. (SF5)

Other SF representatives said they see no point in specifically focusing on sexual abuse, especially since it seems rather uncommon compared to other social concerns or to child protection more broadly.

A few of the SFs seem further along in their work for safe sports, especially the Equestrian Sports, Handball, and Gymnastics Federations have implemented measures to ensure safe sports. A representative of the Swedish Equestrian Sport Federation talked about their work “safe in the stable” (Sweden's many public “riding schools” engage thousands of children, predominantly young girls):

Our starting point was hierarchies in the stables. You know, horse groomers in stables and different [status] levels and so on. In 1997 we adapted “Sports wants” to “Equestrian sports wants”. We then initiated “Safe in the stable”, which is ongoing, focusing on children and youth by talking about healthy leadership, anti-harassment, bullying, and so on. […] The first reported case of sexual abuse was early 2000. Now our sports foundational values are included in our federation by-laws, which means that all clubs must follow the principles and values of the federation. It gives us better opportunities to act.

The Swedish Handball Federation was also relatively early with safe sport measures:

Our Handball Federation safe sport council consists of a board representative, a social worker, a lawyer, and our secretary general. We also have two co-opted persons as resources when needed, a communicator and a psychologist. Our very first step was taken in 2012 at a conference. We wrote a concept document to encourage sports-clubs to develop policy and action plans to manage everything included in our zero vision for our sport.

The Equestrian Sports and Handball Federations address sexual abuse (or harassment) more frequently and explicitly than the other SFs. The Gymnastics Federation directs their measures toward the promotion of sustainable leadership and other positive values instead:

In 2014 a decision was made to initiate a developmental model, a code of conduct, a Gymnastics Ombudsman, and a legal, disciplinary board. […] The risk when listing specifics; does it mean that everything not on the list is okay? So, we don't talk sexual harassment explicitly today, no. We talk about sound leadership. The phrase sexual harassment is not mentioned, not yet anyway, but it's the same behavioral pattern and distribution of power. Or rather abuse of power.

Safe sport measures can be related to several areas and be organized within different departments within the SFs. Measures are neither always termed “safe sport”, nor constitute a specific department, dedicated group, or responsible person within the SF. Safe sport measures were, at the time of writing, particularly uncommon in the SF for football, ice hockey, floorball, and tennis. Examples of measures against sexual abuse that were brought up during the interviews included policy and guidelines, criminal record and reference checks, license and certifications for coaches, education, Sports Ombudsman, disciplinary bodies, and case management procedures. These measures are described next.

#### Policy Documents and Written Guidelines

Regulatory documents and guidelines such as policies, codes of conduct and ethics, checklists, action-plans and so on can outline foundational values and serve as resources for prevention and intervention measures for governing bodies. The Swedish sports movement has documents of their foundational values of sport (“värdegrund”) for the sports movement as a whole and each individual sport and its SF, which is supposed to inform all sports activities. The SF representatives were asked about policies or guidelines that addressed sexual abuse that they had produced or used. All 10 SFs have foundational values for their sport including being healthy, democratic, safe, and positive. These values typically clarify issues such as anti-doping, fair-play, equality, and child rights. Only a few SFs mention sexual abuse explicitly in these foundational values. Policies and guidelines against sexual abuse are even more unusual. None of the SFs have a policy against sexual abuse in addition to the SSC's policy that broadly applies to all sports in Sweden—even though the SSC urges SFs to develop their own sport specific guidelines for a course of action against sexual abuse (SSC, 2005). Some SF representatives say they prefer to refer to the SSC policy: “If the SSC have a policy, we prefer to use that instead of creating our own” (SF9). Others would like to form their own policy against sexual abuse: “We need our own policy for our implementation, I can't see implementation without policy” (SF6). The same applies to guidelines or action plans to prevent or manage sexual abuse; most SF representatives refer to the document “Create safe sport environments” (SSC, 2018) produced by the SSC. These guidelines address bullying, harassment, abuse, and violence (sexual and other forms).

A few of the SFs have codes of conduct, in most cases for coaches and other sport leaders. The Swedish Basketball Federation addresses sexual abuse explicitly and in depth in its code of conduct, which was published in 2020 and applies to everyone with a duty within the SF, including players, coaches, and directors (The Swedish Basketball Federation, 2020). The Athletics Federation and The Swimming Federation have codes of conduct for their national teams that state that sexual abuse and harassment is unacceptable.

To conclude, regulatory and foundational documents and guidelines that address safe sport in general and sexual abuse in particular are sparse. Few SFs have their own policy, guidelines, or bodies to build a systematic foundation for safe sport measures, even less so to address sexual abuse within their sports. These measures seem particularly unusual in the SFs for football, ice hockey, floorball, and tennis.

#### Criminal Record and Reference Checks

The SF representatives welcome criminal record checks for all clubs and SFs. They pointed out that some of their clubs performed criminal checks several years before it became mandatory in 2020. The response from the clubs, however, have been mixed. This raises questions about the implementation of the rule. One SF representative points to the lack of clear guidelines to inform practice in particular matters, for example “if someone has a criminal sentence, that person may be able to stay in the sport club” (SF3). Some SF representatives are worried about an over-reliance on these checks and their ability to detect perpetrators and prevent abuse since “they are only an indicator of criminal conviction history” (SF8). The same SF representative thus advocates for reference checks when recruiting coaches and sport-leaders: “We encourage clubs to check references once a year, that's more important than criminal checks” (SF8). However, reference checks are not mandatory and most likely not routinely implemented by SFs or clubs.

#### License and Certification for Coaches

SFs offers a variety of license, certification, and diploma systems for sport coaches. Formalized license systems for coaches seem to be developed and practiced in some sports more than others. All SFs in the study have at least some sort of requirements for coaches, more so on higher performance levels, albeit not necessarily a formal license system. One SF representative said: “Unfortunately we have no license system for coaches in place, enabling us to pull someone's certification and can show someone's educations and qualifications” (SF6). Coach licenses are usually connected to certain requirements and qualifications, especially regarding education. Even if a SF has no official license in place, coaching courses may be mandatory; for example, a type of “step one course” for basic coaching, and additional courses for higher performance levels. Licensing systems can also be used for disciplinary measures, i.e., if license requirements are not met. If a coach misbehaves for example, his or her license can be temporarily suspended. According to the SF representatives accounts, coach licensing systems are a step toward increased professionalization of sport and sports coaching, as well as toward the increased need for control, competence, and qualifications of coaches more broadly.

#### Disciplinary Bodies

All SFs in the study have some sort of function or person with the role, task, and competence to manage disciplinary and legal cases within their sports. For example, disciplinary boards may employ a group of people or a single legal advisor or lawyer whom the SF consult. Disciplinary measures may concern events that violate SF's rules and policy, employment matters, and so on. One SF representative exemplifies: “I know one case where a coach was suspended and his license withdrawn by the disciplinary board before he was convicted, which he later was” (SF4). Disciplinary measures may also lead to investigative reports of inappropriate behavior or violations of regulations or ethical principles and the determination of disciplinary sanctions. SF representatives who have experience with managing sexual abuse cases note that these types of disciplinary cases, and their typically ambiguous boundaries, tend to be complicated and sensitive.

#### Sports Ombudsman

A new position in Swedish sport is the Sports Ombudsman, who serves as a first point of contact for disclosure or questions regarding sport related concerns. The Equestrian Federation has a Member Ombudsman (currently shared by two appointed persons) and the Gymnastics Federation has a Gymnastics Ombudsman. The SF representatives described these functions as:

The Gymnastics Ombudsman was launched in 2014. Currently it equates a 20 percent employment by an external person. So, the person holding that position has no other role within the federation. Most work consists of receiving reports or questions through telephone or email and refer people. Usually, the Ombudsman doesn't manage or investigate cases, but passes them on within the organization or perhaps outside, like if it's a police matter.

The Equestrian Sport Federation board reckoned we needed a Member Ombudsman function. It was during the same time the SSC discussed a national Sports Ombudsman. Most calls don't concern sexual harassment or such concerns, but more questions about employment legislation and like…. interpersonal matters. Support in deciding what kind of issue and how to move forward according to our regulations.

#### Education

According to the SF representatives, matters like the foundational values of sport, leadership, and athlete welfare are common in coach and leader education nowadays. They point to increased awareness regarding mental health, ethics, social sustainability, and an interest to educate accordingly.

We have included the foundational values of sport and safe leadership in our basic course for many years, which is mandatory for every coach, leader, director, and board member. (SF4)

It seems uncommon, however, for educational content to address sexual abuse specifically and in depth. “So far it hasn't been a specific topic in our educations, but sound leadership and safe environments [is]” a SF3-representative said for example. The need to teach people about sexual abuse specifically is not needed at this point according to some SF representatives. Others point to a lack of competence within their organization to offer such education. Again, the SFs for equestrian sports and handball are exceptions by addressing sexual abuse explicitly. The Handball Federation recently received financial support from the SSC to teach district sport federations about “subtle signs” (e.g., grooming). The youth section of the Equestrian Federation has, as mentioned earlier, created a toolkit called *Safe in the stable*, containing materials and products to use for education, information, and conversation for children and youth and their coaches. It includes cooperation and values exercises, a boardgame, and written cases and stories about sexual harassment and other topics, directed to different audiences and ages to initiate discussion. The Gymnastics Federation has written material for coaches to use for information and discussion with young gymnasts about safe sport topics, including definitions of sexual harassment and abuse.

#### Website Content

The review of the SFs websites indicated that sexual abuse, in any type of wording, is rarely addressed or showcased. Three of 10 websites had content addressing sexual abuse explicitly, usually included under the rubric of safe sport. When sexual abuse was addressed as a subject matter though, it focused almost entirely on sexual abuse of children. The hashtag #metoo was sometimes used as a way to address sexual abuse. Seven of 10 websites addressed safe sports. The review gave the impression of safe sport an unestablished area of work in most SFs, yet more viable to showcase compared with sexual abuse. The contrast is probably due to more positive connotations toward safe sport than toward sexual abuse. There seems to be a resemblance between safe sports measures more broadly and website content addressing sexual abuse and safe sport. It was unusual to find content that addressed children or young people directly. More commonly, content regarding safe sports was directed to adults from a leadership perspective. Content addressing safe sport was often in the form of hyperlinks to SSCs safe sport webpages, although several of these hyperlinks did not work. The findings are summarized per SF in Table 2.

**Table 2:** Findings of SF websites review.

**SF**	**Sexual abuse and safe sport content**
Athletics	Webpage with information about safe sport protocols, accessible through safe sport banner on the frontpage.
Basketball	No content found.
Equestrian sports	Distinguished accessibility, display, and quantity of safe sport content. Several webpages about safe sport, including contact details of their Ombudsman. Addresses sexual abuse explicitly.
Floorball	Webpage with information about safe sport protocols.
Football	No content found.
Gymnastics	Written protocols (e.g., code of conduct) regarding ethical and safe leadership and contact details of their Ombudsman.
Handball	Webpage with information about safe sport and their safe sport council, accessible through safe sport banner on the frontpage. Addresses sexual abuse explicitly.
Ice hockey	No content found.
Swim sports	Webpage addressing sexual abuse with summary of SSCs policy against sexual abuse.
Tennis	No content found.

#### Case Management Measures

The procedures and practices for managing disclosure of sexual abuse differ between the SFs. As mentioned previously, the Gymnastics and Equestrian Sports Federations have ombudsmen appointed to receive reports and the Handball Federation has a safe sports council. The other SFs in the study do not have an official function or appointed person to facilitate disclosure or procedures for case management according to the SF representatives. Hence, disclosure of sexual abuse, misconduct or inappropriate behavior reaches the SF in different ways. Some SF representatives refer these calls to the SSC's whistle-blower or their national Sports Ombudsman. Other SF representatives refer to child protection organizations. According to the SF representatives, disclosure of sexual abuse is rare compared to other types of reported problems. Here are some quotes from the interviews about disclosure and case management:

Nowadays the SSC sometimes contacts us with reports they have received, usually with a person's name or club. Sometimes the case is handled entirely by the district federation and the club in question and not by the sport federation at all. (SF9)

Since we are quite a big sport, cases are talked about. Our general secretary has been contacted, our press department receive calls, our lawyer…. but otherwise, I guess the section it concerns is notified. If it regards a crime, it's the parents' decision, depending on the age [of the athlete]. (SF2)

We encourage clubs to have a contact person to receive calls. If someone doesn't want to contact that person, or the sport federation, there's the SSC whistle-blower function. We also try to encourage notifications of concern for a child to the social services. (SF8)

In the next section, the SF representatives' experiences of managing reported cases within their sports and SF are described.

### Experiences of Case Management

Some of the SFs had reported cases of sexual abuse within their sports. Representatives from six of the 10 SFs says one or more cases of sexual abuse toward children and young people had been disclosed within their sport. Some of these cases are publicly disclosed testimonies from sexual abuse survivors, including a few high-profile cases. Other cases were not publicly dealt with but managed in confidence within the SF or sport-club. Most of the reported cases, however, are described by the SF representatives in terms of ‘inappropriate behaviors' rather than sexual abuse, i.e., cases that do not seem to violate sport policy or regulations. These ambiguous gray-area cases are complicated for the SFs to manage—both *if* and *how* to act as sport federation and administrators. Ethical dilemmas, without guidance by policy or codes of conduct, tend to give rise to conflicts within sport environments. Here is how some of the SF representatives describe their experiences:

The reported cases usually concern inappropriate contact in social media between sport leaders and athletes. It happens time and time again. These cases are complex because it is through rumors and gossip. Thus, we must find a certainness and soundness in this…. not be ruled by some mob mentality. If someone questions why [a leader/coach is suspended] we explain that we have received a report and decided that the behavior doesn't match our foundational values. (SF6)

The cases have all included men with younger women. Like ‘come to my room and sleep in my bed' and the girls agreed. […] We had a case within the national team where a former girlfriend [of a coach] years later claimed to have been abused. It is hardly an unlikely situation to occur. (SF4)

It's common with cases that concern rumors about coaches or other leaders who form relationships with athletes, sometimes minors. Like inappropriate behaviors but not abuse. And if you ask them, everybody denies it. I mean, how do you deal with that? In our sport there are coaches who have been in that gray-area and we have no system or structure to stop them. (SF10)

According to the SF representatives, case management varies in many regards. For example, how the disclosure or reporting took place, how the reports were received, how (or if) the cases were managed by the SF, and the long-term and short-term impact of the disclosure. Reported cases of sexual abuse that led to public scandal seem to prompt SFs to act. In four of the SFs, no cases of sexual abuse had been disclosed. The same four SFs have taken no measures against sexual abuse and few measures for safe sports overall. Representatives from these four SFs believe that one reason why protection measures have not been prioritized in their sports is because they have had no known cases of sexual abuse. In a few SFs reported cases of sexual abuse seem to have served as wake-up calls or starting-points for safeguarding measures. For example:

If it [sexual abuse] can happen in such well-renowned club, it could happen anywhere else too. We realized we needed a comprehensive strategy to work proactive and reactive if something happens. So that case also had an impact on how we started to take action for safe sports. (SF8)

In most of the SF representatives' experiences, however, the measures taken only pertained to the reported cases in question, i.e., damage control and crisis management:

After that [high profile] case, written guidelines were formulated. But that was mainly a crisis response. Coming up with something, but without any plan on how to move forward. (SF10)

Managing reported cases of sexual abuse, including accusations, rumors, and ambiguous boundaries can be challenging for the SFs. At the same time, having had these experiences can help develop future measures:

In the beginning, before, we were lost. Less wise. Like how we responded in the media and probably appeared quite unorganized. Now we are slightly less worried about what's written. […] Crucial for our measures are wise board-members, especially during our crisis. Who dared to touch on the subject? We have expanded our competence and approach this as a matter of quality. We have the awareness and governing bodies to turn to in need. (SF1)

### Facilitators and Barriers for Implementing Sport Policy Against Sexual Abuse

These results may raise questions about why so few protection measures against sexual abuse has been taken by most of the SFs to date. It can also be valuable to explore what may facilitate and motivate SFs and other stakeholders to cultivate such measures and develop a safe sport culture. The SF representatives' accounts regarding both facilitators and barriers to implement sport policy against sexual abuse are presented below.

The one facilitator that was emphasized by all SF representatives was the fundamental principle that child sexual abuse is completely unacceptable and that a safe sports environment for children is of crucial importance. One SF representative put it like this:

These are issues that have never been brought up before but that should be obvious. Good leadership and safe athletes; a safe sport environment is key to becoming a good athlete. (SF6)

The incoherence between these statements and the SFs lack of actions to cultivate safe sport environments is obvious. Another distinction that can be noted is between the increased attention to sexual abuse during recent years and, simultaneously, awareness of the scandals and crises it can evoke. This awareness relates to the existence of sexual abuse in sport and the sensitivity and social stigma related to it. These two sides of the same coin are thus likely to create a conflict of interests. One SF representative said, for example: “If the sport isn't perceived as safe, you simply won't attend, it's that simple, [it's] an important part of the brand nowadays.” At the same time, if negative publicity surfaces, such as a sexual abuse scandal, it is likely to hurt the sports trademark. Another SF representative says: “You protect your own and silence anything that may tarnish your club, sport, or brand.”

Another important barrier against implementing protection measures that was mentioned by all SF representatives is the lack of resources, especially on a club level. Swedish sport clubs typically have limited human, financial, organizational, and educational resources. These limitations may be amplified by a disconnect between the local, district, and national levels; for each level the implementation of sport policy tends to weaken. SF representatives said:

It's always a balancing act between work and administration. Criminal record checks are not a big thing, but for a small club with all volunteers, it becomes another task added to the pile of work. (SF5)

Sports clubs depend on volunteers. Often driven individuals who carry the whole club. They are both board-members and do operational groundwork. Thus, as sport federation we must provide clear frameworks that reach the clubs. (SF6)

The measures must function on a local club level because that is the foundation of sports. Otherwise, a gap is created between the SSC and the federations. And between the SSC's foundational values and written policy to getting the sports movement to implement these. Currently, this gap is huge and that is a problem. It's easier to take good initiatives than what the clubs manages to achieve. Also, these relatively new questions on safe sports requires knowledge and resources we don't yet acquire. (SF2)

The limited resources necessitate priorities. The SF representatives say that safeguarding sports seldom leads to perceivable results. Especially not when preventative, long-term safeguarding measures are compared to striking results from performance achievements in competition. Prioritizing or incorporating protection measures in routine sport activities can thus be perceived as a thankless task, perhaps to no avail, and thus impracticable. Moreover, typical “sports activities” concern physical, technical, and tactical training sessions, competing, and so on—not engaging in safe sports environments. The SF representatives continue:

Our focus has been to become the best at our sport, not on having the best environment. (SF3)

We don't have the routines to address these types of questions [safeguarding]. Had it concerned competition, or physical achievements, then these gaps would not exist. It would have been handled immediately. (SF2)

The main problem working with values and social sustainability is the priority to bring forth our next big stars. You focus on results. (SF7)

Although the barriers that the SF representatives address apply to safe sport measures in general, the results indicate that measures against sexual abuse can be even more challenging because it is particularly sensitive and taboo:

This matter, sexual abuse and harassment, is special. Much more shrouded in silence. I mean, nobody wants to be seen as racist either, but you can still talk openly about racism. I can talk to a journalist about racism in our sport, but sexual abuse is much more difficult. (SF6)

The problem is partly that you cannot mention sexual abuse and what that entails clearly and direct, partly that you cannot manage these questions or cases in a straightforward manner. During #metoo we got calls from parents in other sports saying they could not speak about this, that the language and dialogue was non-existent and unthinkable. (SF10)

According to the results, sexual abuse is typically addressed only when cases have been made public. A few SF representatives have experienced that initiating measures against sexual abuse can in itself spark speculations and worry that something bad has happened. As one SF representative put it: “I'm quite sure abuse has occurred, but our sport gets away [with it] because the culture of silence doesn't leak.”

There are also other barriers brought forward by the SF representatives are certain attitudes and norms that may contribute to unhealthy environments and to enable or camouflage abuse:

The macho culture in our sport runs from coach to athlete, making you less receptive to new knowledge, and depict these questions as unimportant. Also, a competition logic hampers development. Sharp elbows and expectations of a tough environment, performance at all levels and thus corresponding behaviors. (SF3)

Some SF representatives say that the social status of a leader or coach can sometimes enable them to act as they please, despite rules and policy. “The status of a successful leader or coach is pivotal; your word counts no matter what you've done,” a representative from SF6 says for example. Another SF-representative says: “A colleague once advised me that ‘sometimes it's better to not say what you think” (SF10). Others describe how close-knit friendships and family-bonds that demand loyalty may also contribute to the notion of “protecting your own” by keeping quiet.

Lack of knowledge and awareness was often mentioned by the SF representatives as a common problem to the implementation and development of protection measures. More specifically about what should (or could) be done against sexual abuse, as well as the training and experience to execute such measures, present a great challenge for the SF. Safe sport is an area of work that is new to most of the 10 SFs. Sexual abuse is rarely addressed in education, even though they have started to address ethics, foundational values, child rights, mental health, sustainable leadership, and similar topics more frequently than before. In addition, some participants describe a distinct reluctance to acknowledging sexual abuse as a problem in sport—and in their own sport in particular:

I believe there is a lot of ignorance and a blind eye toward it; ‘I had no idea….'. But I don't buy it, that no one had heard nothing about those cases. We are a part of society, no free zone. The question is rather that you don't want to know. (SF6)

There is a resistance. Not like no one gives a damn, but as in taking actions and work…. ‘such fuss, of course we are safe', stuff like that is common. ‘Milk and cookies' [degrading words] some call it. (SF7)

Soon before #metoo I addressed sexual harassment and abuse. And the response I got, or the consensus was that it doesn't occur in the same extent as in society at large. That sport is much better. After #metoo though, it was easier to get a response. […] But some believe you can solve this by using a label like #metoo, write a bit about it, and that's it. Like ‘throw in a #metoo-thing and check that off the list'. (SF10)

To conclude, several barriers that the sports movement must address and overcome to implement sport policy against sexual abuse was identified in the analysis. A fundamental barrier is the culture of silence, which hampers the development of protective measures and may enable and camouflage sexual abuse. Sexual abuse, more so than any other matter according to the results, is perceived to be so sensitive that it sparks a fundamental conflict between breaking and maintaining the culture of silence through socialization within sport.

## Discussion

In the following section I will discuss challenges for developing safe sport and how such problems can facilitate abuse and debilitate protection measures. First, I discuss the motives, focus, and forms of the SFs measures against sexual abuse. Second, the culture of silence and conflict between making sexual abuse visible or invisible is discussed. Third, implementations for developing sexual abuse policy into practice is discussed, including the contextualization of sexual abuse in sport.

### The Motives, Focus, and Forms of SFs Measures

The analysis indicates that the same two or three SFs (for equestrian sports, gymnastics, and partly handball) distinguish themselves for having taken more safe sport measures and for a longer time period. The results neither reveal any distinct, categorical explanation why these particular SFs have taken more measures against sexual abuse than others, nor why some SFs (for football, ice hockey, tennis, and floorball) had basically no safe sport measures in place. The more engaged SFs have had reported cases of abuse within their sport and public disclosure of sexual abuse *can* function as a starting-point for safe sport measures—but not necessarily. For example, Sweden's most noticed and consequential high-profile case of sexual abuse to date (the disclosure by Patrik Sjöberg) happened in athletics, but the Athletic SF seem to have very few measures against sexual abuse. According to some SF representatives, all too often disclosure of sexual abuse has only resulted in “damage control” of the particular case and sometimes no measures has been taken at all.

Besides from the existence of safe sport measures, the framework and organization of measures also deviate between the SFs. As outlined in the introduction, Swedish sport has broadly been influenced by a values-based framework (cf. Gurgis and Kerr, 2021). Such a framework was particularly prominent in the Gymnastics Federation's measures, promoting positive values and sustainable leadership. Having that said, the public attention to child sexual abuse in sport during recent years seems to have prompted a prevention of harm and protection of children framework (cf. Gurgis and Kerr, 2021). The measures that have been initiated by the SFs in recent years seem to be directed toward detecting, reporting, and managing cases of misconduct, harm, and abuse toward young athletes. Reactive measures are important, but researchers have raised concerns about an over-reliance on systems of disclosures and sanctions to deter abuse because of the under-reporting and because it tends to place the onus for abuse prevention on the subjected, vulnerable individuals (often the athletes) (Mountjoy et al., 2016; Solstad, 2019; Komaki and Tuakli-Wosornu, 2021). This stresses the importance of measures to prevent sexual abuse and promote a safe sport culture. Unfortunately, prevention measures seem more difficult for SFs to implement. Safe sport is a separate, downplayed issue on the side, at best, while “real” sport activities comprise training sessions and competitions. Addressing sexual abuse is particularly problematic because of negative connotations and sensitivity, judging by the SF representatives' responses. Thus, there is little positive reinforcement of measures against sexual abuse in practice (cf. Komaki and Tuakli-Wosornu, 2021 “carrot and the stick” analogy for positive and negative reinforcement of behavior in sport organizations).

Meanwhile, a distinguished motivator for measures against sexual abuse found in the study was the unanimous statement, emphasized by all SF representatives, that sexual abuse in child and youth sport is absolutely unacceptable. Despite this consensus though, there is a distinct discrepancy between this principle and the implementation in terms of actions. Thus, the strongest motive is also connected to the greatest challenge—the implementation of policy. This conflict is discussed next.

### The Culture of Silence

A fundamental barrier to the implementation of sport policy against sexual abuse that became apparent in the analysis was an underlying conflict between making sexual abuse visible or invisible—which is currently in favor of the latter. Although many studies confirm a high prevalence of sexual abuse in sport, it is common for it not to be disclosed (Bjørnseth and Szabo, 2018). This may contribute to a sense that sexual abuse is uncommon in the sport context in a “no news is good news” manner. The relatively few cases brought to light, especially high-profile cases, tend to play out as scandals and crises for the sport in question. This became particularly apparent in Sweden during #metoo.

Consequently, addressing sexual abuse is not necessarily associated with solving problems and promoting safe sport, but to potentially *cause* problems and inflict fear and a sense of unsafety. From this perspective, sport organizations likely benefit from keeping sexual abuse invisible and reinforcing a culture of silence in sport. In connection, there is a potential conflict of interest and roles at play here given the self-governance (including monitoring and regulation) of sport (e.g., Kerr et al., 2020). Eliasson (2017) points to a “conflict between the Swedish sports movement's decentralized governance model of autonomy for membership organizations and the ability of the SSC to sustain the child rights perspective in Swedish child and youth sport” (p. 473). The social factors of sport also come into play, including the often-close-knit relationships of power, trust, and loyalty that is mentioned by the SF representatives. Power imbalance in sport is one of the most influential factors for enabling sexual abuse and the status quo of sport (Roberts et al., 2020). Mountjoy et al. (2016) points out that “Sexual harassment and abuse in sport stem from abuses of power relations facilitated by an organizational culture that ignores, denies, fails to prevent or even tacitly accepts such problems” (p. 1020).

Arguably, these circumstances foster inactions and ignorance that reproduce social and organizational tolerance for sexual abuse. This is manifested by the gap between policy and consensus against sexual abuse, and the implementation of policy and principles into practice. The culture of silence thus poses a fundamental challenge to prevent sexual abuse in sport (as in society at large) and the achievement of substantial, systemic change. I will continue to discuss the conflict of interests and problems that can prevent the development of policy into practice next.

### Developing Policy Into Practice

The discussion about the (lack of) measures against sexual abuse raises questions on how to implement policy into practice. Besides the social factors discussed in the previous section, several organizational factors that can debilitate safe sport measures were found in the study. Due to the governmentality and organization of the Swedish sport movement, the SSC are in a weak position to control the implementation of policy against sexual abuse in relation to the autonomous SFs. In turn, SFs have little control over how sport-clubs are led, and they cannot enforce good safe sport practice among individual stakeholders (cf. Barker-Ruchti et al., 2018). For example, criminal record checks of sport-leaders in all SFs and sport-clubs are one of few requirements by the SSC. However, there is no procedure in place for the SSC or the SFs to monitor if or how these checks are performed. Drawing on the theoretical framework, a holistic, whole-of-system approach is advised to meet the diverse, multilevel challenges that is required to promote safe sport and prevent sexual abuse (cf. Rhind et al., 2017; Roberts et al., 2020).

Including sexual abuse in a larger systems approach to safe sport also makes sense given the diversity and ambiguous boundaries of sexual abuse and “inappropriate behaviors” that the SF representatives addressed. Ambiguous boundaries between appropriate and inappropriate behavior and sexual abuse pose dilemmas and problems (to define, regulate, and manage) that sport organizations seem particularly ill equipped to manage both in policy and practice (Johansson, 2013). It is possible, for example, that what is initially perceived or reported as “inappropriate behavior” may later be disclosed as abuse (Johansson, 2017; Gaedicke et al., 2021). Brackenridge (2001) wrote that “between the extremes of behaviors from those that were definitely acceptable to those definitely constituting sexual harassment there were [are] many context-dependent ambiguities” (p. 55) and gray-areas “give rise to the most pain, both for individuals and for organizations, since [they are] the most difficult area to respond to and control” (p. 207). In connection, a more holistic contextualization of sexual abuse, sexual relationships, and related gray-areas has also been called for in previous research to inform sport policy and practice (Bringer et al., 2002; Johansson, 2013; Gaedicke et al., 2021).

In addition to the need to incorporate prevention of sexual abuse into the larger cultural and organizational context of safe sport, the present study demonstrates that at times specific and specialized measures to target sexual abuse may be called for. A fundamental reason to explicitly address sexual abuse is the silence and sensitivities that can be particularly consequential regarding sexual abuse. The SF representatives voiced a reluctance to initiate conversations about sexual abuse, and uncertainty about *how* because sexual abuse is perceived as a particularly sensitive and difficult topic. Yet, instead of counteracting these challenges by taking specialized measures, the subject of sexual abuse is avoided. In connection, there seem to be a common reluctance among stakeholders in sport to (publicly) recognize sexual abuse as a prevalent, structural problem—especially in one's own—that also exist in Swedish sport (cf. Hartill, 2014; Mountjoy et al., 2016).

In view of the above, I suggest that a systems approach to safe sport incorporates specific and explicit measures to target certain problems at times, in this case sexual abuse in child and youth sport. There is a gap between sexual abuse policy and practice that requires multiple measures to aid and enforce good practice in sport organizations on national and local levels. Given the sensitivity of sexual abuse and the culture of silence, I suggest that this challenge must be taken on a shared, coordinated endeavor by the SFs and the rest of the sports movement.

## Conclusions

Through a sociological lens on sport as a social and cultural institution, this study aimed to explore measures against sexual abuse by the 10 largest sports federations (SFs) for child and youth sport in Sweden. According to the findings, measures to implement sport policy against sexual abuse are sparse. Most SFs have no particular measures that address sexual abuse, including the website content. Safe sport measures more broadly have started to be developed with a few particular SFs in the forefront, whereas others are in the initial phases of organizing safe sport measures. The safe sport measures also vary in regard to conceptualization and organization between SFs. Hardly any of the SFs have a dedicated department, team, or staff within their organization with safe sport as primary responsibility.

Protection measures that have been taken by the SFs include criminal record checks, coach licensing, sports ombudsmen, and disciplinary bodies. Thus, most measures are directed toward detecting, reporting, and managing cases of misconduct and abuse. Six of the 10 SFs have had reported cases of sexual abuse within their sport according to the SF representatives. Many reported problems concern “inappropriate behaviors” with ambiguous boundaries. These grey-area cases seem particularly difficult for the SFs to manage due to a lack of both policy and practice.

Measures against sexual abuse are motivated by the SF representatives' clear stance that sexual abuse is unacceptable. However, there is a distinct discrepancy between convictions and implementation. A fundamental conflict between making sexual abuse visible or invisible emerged during the analysis, favoring the latter, contributing to a gap between policy and practice. Reproducing a culture of silence surrounding sexual abuse in sport seems advantageous for the SFs because it keeps sexual abuse out of sight and out of mind on the surface. In conclusion, the intersection of social and organizational factors debilitates safe sport measures and facilitate sexual abuse in sport. Implications for the sport movement to implement policy includes for sport organizations to acknowledge and prioritize sexual abuse as structural problem that requires greater individual, organizational, and social responsibility by stakeholders at all levels.

## Data Availability Statement

The original contributions presented in the study are included in the article/supplementary material, further inquiries can be directed to the corresponding author/s.

## Ethics Statement

The studies involving human participants were reviewed and approved by Etikprövningsmyndigheten, Sverige. The patients/participants provided their written informed consent to participate in this study. Written informed consent was obtained from the individual(s) for the publication of any potentially identifiable images or data included in this article.

## Author Contributions

The author confirms being the sole contributor of this work and has approved it for publication.

## Funding

This study was funded by the Swedish Sport Confederation (Riksidrottsförbundet) and conducted at the Swedish School of Sport and Health Sciences. The publication fee for open access was funded by the Swedish School of Sport and Health Sciences.

## Conflict of Interest

The author declares that the research was conducted in the absence of any commercial or financial relationships that could be construed as a potential conflict of interest.

## Publisher's Note

All claims expressed in this article are solely those of the authors and do not necessarily represent those of their affiliated organizations, or those of the publisher, the editors and the reviewers. Any product that may be evaluated in this article, or claim that may be made by its manufacturer, is not guaranteed or endorsed by the publisher.

## Footnotes

^1^The SSC was not involved in any part of the study besides suggesting the sample.

^2^Floorball is a type of indoor floor hockey played with sticks and a plastic ball. Each team has five players and a goalkeeper.
